# The effects of a mobile-based multi-domain intervention on cognitive function among older adults

**DOI:** 10.1016/j.pmedr.2023.102165

**Published:** 2023-02-24

**Authors:** Junhyoung Kim, Myungjin Ko, Jungjoo Lee, Yongseop Kim

**Affiliations:** a Department of Health & Wellness Design, School of Public Health, Indiana University Bloomington, IN, United States; b School of Medicine, Seoul National University, Seoul, South Korea; c Silvia Health Inc. Seoul, South Korea

**Keywords:** Dementia prevention, Digital health, Cognitive function, Multi-domain lifestyle intervention

## Abstract

Behavioral and social research has stressed the importance of multi-domain lifestyle interventions to prevent, delay, or remedy age-related cognitive decline among older adults at high risk for dementia. In light of the benefits of multi-domain interventions, some studies demonstrate the effectiveness of a mobile-based program for dementia prevention. Unfortunately, there is limited evidence of whether or not a mobile application of a multi-domain program is effective in improving cognitive functioning among older adults who are a high risk for dementia. Thus, the purpose of this study was to determine whether there was a pre-post change of cognition and there were age-group differences in pre-post change after the 12 weeks multi-domain program, *Silvia program* (N = 59). The Silvia program consisted of five domains, including (a)daily smart routine, (b)cognitive training, (c)lifestyle monitoring, (d)home-based exercise, and (e)voice-based AI cognitive assessment. Using a paired *t*-test and analysis of variance, this study found that there was a significant mean difference in cognitive function scores between pre and post-intervention (95 % CI = 1.10–1.87, *P* <.05). The difference score in cognitive function was higher in the old-old group than in the young-old group (95 % CI = -2.29–0.10, *p* <.05). The findings of this study demonstrated that Silvia program was effective in promoting cognitive function among older Korean adults, especially for the old-old group.

## Highlights

• There is a lack of literature on the use of mobile-based multi-domain programs.
• Silvia Program is a multi-domain lifestyle app designed for dementia prevention.
• Silvia Program is effective in promoting the cognitive functioning of older adults.
• This study supports a mobile-based multi-domain program for dementia prevention.

Currently, nearly 11.7 % of adults in the United States older than 65 years old are experiencing cognitive function decline (Taylor et al., 2018), and estimated 5.8 million people are experiencing Alzheimer’s disease and related dementias (ADRD) (Alzheimer’s Association, 2021). Cognitive function declines with aging and is likely to cause subjective cognitive decline (SCD) and mild cognitive impairment (MCI). As people age, they face a higher risk of declining cognitive function and related cognitive disorders (Mella et al., 2016; Salthouse et al., 2012). A preventive approach has been suggested for maintaining and improving cognitive function throughout the aging process and to apply effective interventions that support cognitive performance improvement in older adults (Bruder-Hofstetter et al., 2018, Myers et al., 2018, Yoon et al., 2018, Reijnders et al., 2015).

Behavioral and social research on cognitive decline and ADRD stresses the importance of non-pharmacological interventions that can address the multiple risk factors of older adults who are at high risk for ADRD (Kivipelto et al., 2018a, Kivipelto et al., 2018b). Previous studies that have investigated the effectiveness of single domain interventions, including exercise, cardiovascular health measures, diet, and cognitive training, have yielded mixed positive outcomes in large randomized clinical trials. However, many of these single-domain interventions have been found to be ineffective (Chow et al., 2002, Guo et al., 2020, National Institutes of Health, 2017, NIH RePORTER, 2017) in clinical trials with small samples and methodologically limited approaches, raising questions about their effectiveness for dementia prevention among community-dwelling older adults and suggesting the importance of multi-domain programs approach for achieving better health outcomes for this population.

The Finnish Geriatric Intervention Study to Prevent Cognitive Impairment and Disability (FINGER) is considered the first clinical study to have used a multi-domain non-pharmacological approach to investigate the prevention of cognitive decline and related disabilities among older Finnish adults with high risk for dementia (Kivipelto et al., 2018a, Kivipelto et al., 2018b). This approach includes attention to exercise, diet counseling, vascular risk management, social activity, and cognitive training. In the FINGER study it was found that participating in a multi-domain lifestyle intervention reduced older adults’ risk of dementia (Kivipelto et al., 2013). Multiple studies have adopted the FINGER study as a platform for investigating the effectiveness of multi-domain lifestyle interventions to prevent or reduce cognitive decline (Lehtisalo et al., 2022, Ngandu et al., 2015), which proved to be effective in preventing or reducing cognitive decline among older adults.

While multi-domain interventions for dementia prevention have significant merits, the primary drawbacks of face-to-face interventions in real clinical settings are the intensive monetary, time, and space resources required and the challenges of monitoring all participants’ adherence to the program (Essery et al., 2021). Research suggests that advanced technologies and Artificial Intelligence (AI) can play an important role in cost-effective, accessible delivery of cognitive training that is individualized, immersive, and engaging for users (Essery et al., 2021, Bohr and Memarzadeh, 2020). Prior studies support the idea of the effectiveness of digital health platforms accessible through mobile-based programs as an intervention for dementia prevention (Eggink et al., 2021, Hafdi et al., 2021, Richard et al., 2019). Clinical trials involving these platforms demonstrate their potential to improve cognitive functioning among community-dwelling older adults in the United Kingdom, Japan, and China. For example, using the internet and mobile phones has been found to reduce depressive symptoms and support self-care management in patients with dementia (Brown et al., 2019, Guo et al., 2020, Liao et al., 2020). The features of the apps of primary value to this population include providing reminders and notifications, giving healthcare tips, and enhancing social networks (Brown et al., 2019, Guo et al., 2020, Liao et al., 2020). However, more scientific evidence of how a digital health intervention can lead to enhanced cognitive function among people at high risk for dementia is needed.

The purpose of the present study was to investigate the health benefits of multi-domain interventions and digital health interventions, specifically, the effects of a mobile-based multi-domain lifestyle program on the cognitive functioning of older adults at high risk for dementia. In light of the World Health Organization’s (WHO’s) emphasis on the need for testing the feasibility and efficacy of such multi-domain lifestyle interventions in different geographical and cultural settings (World Health Organization, 2019), we selected older Korean adults as a sample. Also, there is a lack of literature on the use of mobile-based multi-domain lifestyle programs on community-dwelling older adults living in East Asia. In this study, we collaborated with the Silvia Health company, a digital health company specializing in dementia prevention, which offers a mobile-based app, the ***Silvia program***, that provides cognitive health care for smartphone-owning older adults between the ages of 60 and 89 who show signs of dementia. The program’s components address diet and nutrition, home-based exercise, cognitive activities, voice-based AI-led cognitive assessments, and sleep quality and patterns. Silvia Health designed and developed this multi-domain lifestyle app based on evidence-based therapy programs in collaboration with public health researchers and clinical psychologists.

Thus, the objectives of this study were as follows:

1. To determine whether there was a pre-post change in cognition after participating in the 12- week Silvia program
2. To determine whether there were age-group differences in the changes in cognitive function (e.g., the gap between pre and post) after participating in the 12-week Silvia program

## 1 Methods

### 1.1 Data sample and data collection

This study is a single-arm trial that investigates the effect of the 12-week Silvia program (provided in Korean) on the cognitive functioning of older Korean adults. During the first three months of the project, we contacted local clinics and senior hospitals to recruit study participants representing the target population. The potential subjects were determined as having probable dementia based on a self-reported doctor’s diagnosis of Alzheimer’s disease or dementia. With the permission of the directors of each facility, we distributed recruitment materials to individuals who met our criteria of inclusion and exclusion. Inclusion criteria were being (a) between 60 and 89 years old, (b) able to operate a smartphone/tablet properly, and (c) able to carry out everyday activities without help of others. Exclusion criteria were having (a) a history of dementia or major depression disorder, (b) severe sensory problems associated with sight and hearing, or (c) current involvement in another intervention trial. Once interested potential participants contacted us, we conducted telephone screening to verify their eligibility. Of 72 potential participants who participated in the screening process, 59 met the inclusion criteria. These participants were then scheduled for an intake procedure at the clinics and hospitals. At intake, the research team obtained informed consent, helped participants install the mobile app and demonstrated its use, and asked participants to complete the online baseline questionnaire.

### 1.2 Intervention program

**Fig. 1:**
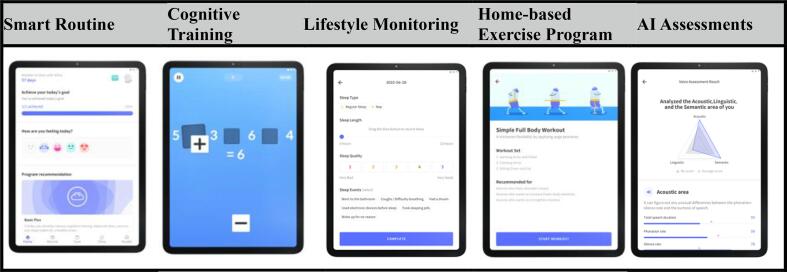
Silvia Program.

The Silvia mobile app was developed by researchers, clinical professionals, and engineers from Silvia Health Company based on the feedback of health professionals and caregivers and on the comments on existing mobile health apps. The app is available for download from the App Store or Play Store. The Silvia mobile app (Fig. 1) was designed primarily for healthy older adults and older adults with MCI to manage and improve the cognitive functioning via multidomain health behaviors approach. The app consists of five main components include (a) daily smart routine, (b) cognitive training (15- to 30-minute sessions three times a week for 12 weeks), (c) lifestyle monitoring, (d) home-based exercise programs (30 min twice a week for 12 weeks), and (e) voice-based AI cognitive assessments. In addition, the participants received weekly and monthly summaries of their activities and access to the results of their cognitive assessments. The program was implemented between July and October 2020.

#### 1.2.1 Smart routine

**Fig. 2:**
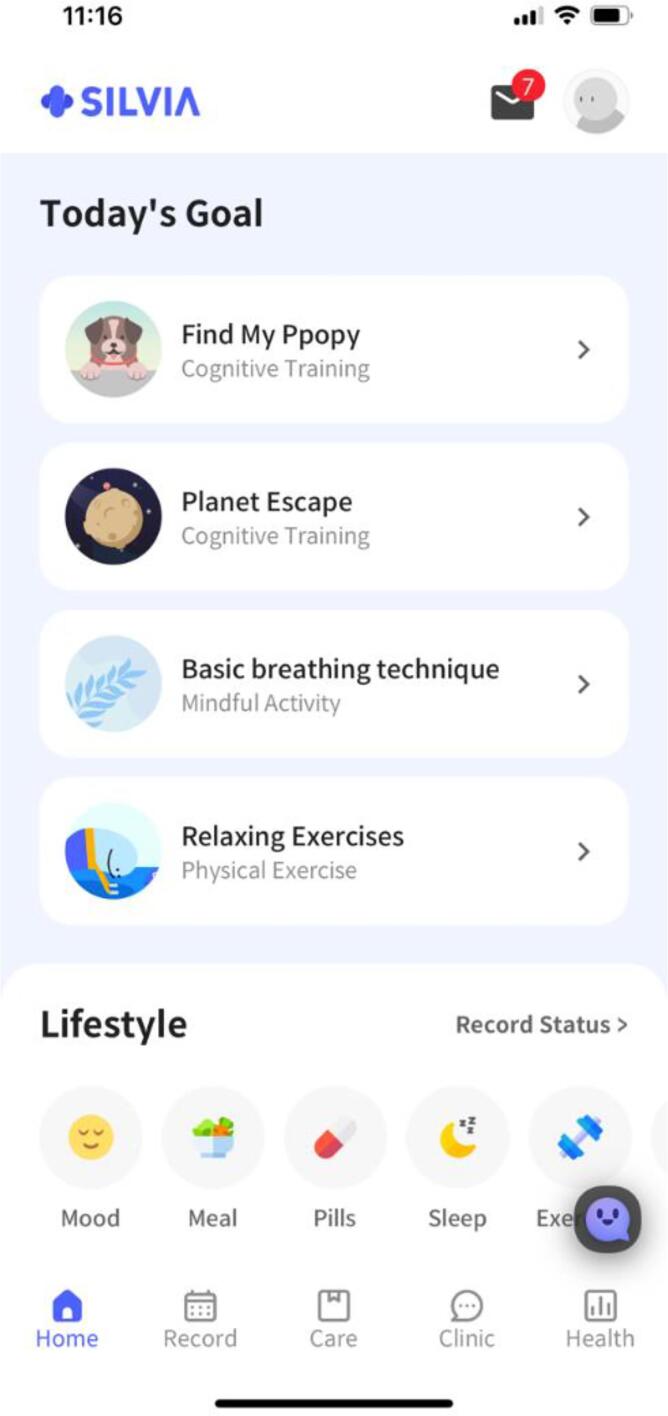
Smart Routine.

This app provides daily routines for users to follow, including diet monitoring, learning activities, social games, memory games, and speed training, as well as brain activity information. As a motivational tool, users received a list of achievable daily activities, and the app sent each user a notification of these activities. The smart routine consists of 6 domains of lifestyle components. For example, users receive a list of two cognitive trainings on the app, mindfulness activity (meditation with narration), physical exercise, and health-related articles each day (Fig. 2).

#### 1.2.2 Cognitive training

The cognitive training component comprises a variety of training contents for memory enhancement, concentration, visuo-spatial awareness, senior executive ability, and linguistic competence. Altogether the app provides users with 15 validated training programs spanning a wide range of cognitive domains, each adjusted to the appropriate degree of difficulty for each user. program. Activities included detecting patterns, memorizing lists, and using a touch screen program to increase cognitive function. Updates occurred weekly. Table 2 provides examples of cognitive training exercises.

#### 1.2.3 Lifestyle monitoring

**Fig. 3:**
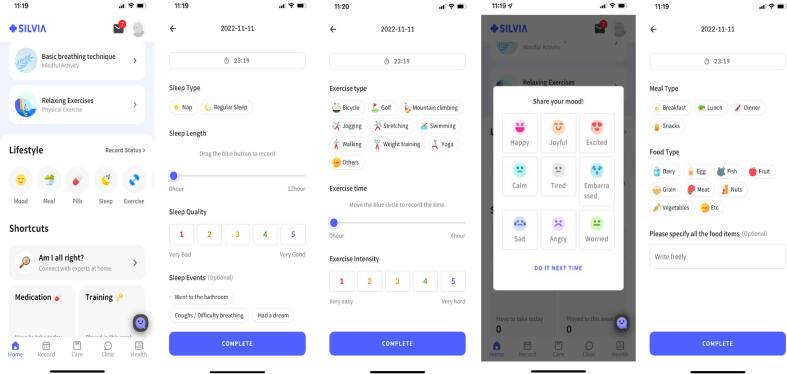
Life Monitoring.

Participants completed self-report assessment about their five lifestyle domains, including utrition, activity patterns/intensity, and sleep patterns/quality (Fig. 3). Nutritional guidance based on the Korean Dietary Guidelines for Older Adults was provided to them each week. Physical activity was assessed based on the types and intensities of daily activities in which participants partook, while social activity was assessed based on types of social context. Participants could track their diet, activity, and sleep patterns based on the data they provided.

#### 1.2.4 Home-based exercise program

Home-based exercises were developed from previous studies (Chen et al., 2020, Cruz-Jentoft et al., 2018). This mobile-based exercise program consisted of two main types: aerobic exercise and resistance exercise and was conducted twice a week for 12 weeks. This was delivered with step-by-step illustrations. Resistance exercise regimens were based on the use of elastic exercise bands. The color of the elastic band was not assigned, and participants chose their routine based on their fitness levels. Each session included a 5-minute warm-up, 10 to 15 min of resistance training (seated row, one leg press, seated leg raise, semi squat, bridging), and 5 min of cooling down. The exercise intensities were set by the color of the elastic exercise band. The aerobic exercise regimens were based on fast walking. Each session included a 5-minute warm-up, 10 min of fast walking, and 5 min of cooling down.

#### 1.2.5 Artificial Intelligence (AI)-led cognitive assessments

Voice-based AI-led cognitive assessments were conducted with each user. Results were automatically shared with users and they reviewed their results weekly. The assessments measured the difficulty of each task, a score for each cognitive exercise, and time spent by each user.

### 1.3 Health outcome measure

To assess cognitive functions, we used the Korean version of the Cognitive Impairment Screening Test (CIST), a revised form of the Mini-Mental State Examination (MMSE), which has been tested for validity and reliability (Baek et al., 2016, Hwang et al., 2022, Jeong et al., 2004). The CIST has been widely used in nursing homes, assisted living facilities, and hospitals in South Korea (Hwang et al., 2022, Lee et al., 2021). The CIST questionnaire is a self-report instrument which requires no more than 10 min to complete. The CIST is composed of 13 items in five domains: general cognition (two items for 5 points), memory (two items for 10 points), attention (two items for 4 points), language (two items for 4 points), visuo-spatial ability (one item for 2 points), and behavioral function (four items for 6 points) with a maximum a total score of 30 points. The CIST is not a medical tool for diagnosing dementia but a research instrument for assessing cognitive functioning.

### 1.4 Statistical analysis

A paired *t*-test (*p* <.05) was used to compare the mean scores of cognitive functions after participants had taken part in the Silvia program (*n* = 59). An Analysis of Variance (ANOVA) was then performed to investigate the mean differences in the cognitive function scores before and after the intervention for each of three age groups (i.e., Young-old, 62 to 68; years old, Old 69 to 75; Old-old, 76 to 88), a classification taken from previous studies (Lee et al., 2021, Lee et al., 2018). The analysis was conducted using the Statistical Package for the Social Sciences (SPSS) version 26.0.

### 1.5 Ethical consideration

We obtained ethics approval to conduct the study from the Institutional Review Board (#15198). Each participant in the intervention and the control groups was informed of the title, objective, duration, and type of the study, and the consent form was read aloud to them, ensuring they understood the objective and content of the study. Written consent was obtained from those who agreed to participate in the study. The data collection and application phases were initiated after consent was obtained.

## 2 Results

The age of the participants ranged from 62 to 88 (M = 72.11, SD = 6.54), and the age groups, Young-old, Old, and Old old, comprised 25, 24, and 23 participants respectively (Table 1). Years of formal education ranged from 1 to 19 (M = 10.02, SD = 3.78). The composite cognitive function scores were 18 to 30 (M = 26.63, SD = 2.86) before the intervention and 20 to 30 (M = 28.12, SD = 2.58) after the intervention.

**Table 1:** Demographics.

	Age					
	62 to 68		69 to 75		76 to 88	
	M	SD	M	SD	M	SD
Age	64.88	2.07	72.49	2.04	79.61	3.32
Education year	11.68	2.99	10.96	3.82	6.72	2.42
Cognitive function (Pre)	28.55	1.36	26.86	2.01	24.06	3.23
Cognitive function (Post)	29.35	1.09	28.64	1.87	26.00	3.34

**Table 2:** A paired *t*-test.

		95 % CI				
	Mean	SD	Lower	Upper	t	Sig.
Pre – Post Difference						
	1.392	1.48	1.10	1.87	7.75	0.00*

* *p* <.05.

A paired *t*-test (Table 2) found a significant mean difference between pre- and post-intervention cognitive function scores (M = 1.49, SD = 1.48, CI = 1.10 to 1.87) (*t* = 7.75, *p* <.05). The result of the ANOVA (Table 3) also showed significant differences between the pre- and post-intervention cognitive function scores across the three age groups (F = 3.69, p <.05). Furthermore, a Bonferroni post-hoc (Table 4) was implemented to investigate differences among the three group mean scores and indicated that the Old-old group demonstrated more change in cognitive scores than the Young-old group (MD = − 1.14, 95 % CI = -2.29, 0.10, p <.05).

**Table 3:** ANOVA result.

	Sum of Square	df	Mean Square	F	Sig.
Intercept	132.06	1	132.06	66.03	0.00
Age	14.74	2	7.37	3.69	0.03*
Error	112.00	56	2.00		
Total	258.00	59			

* R Squared = 0.177.

* *p* <.05.

**Table 4:** Bonferroni post-hoc for change of cognitive score.

Three age groups	Age (I) (Adjusted mean)	Age (J) (Adjusted mean)	Mean Difference (I-J)	Sig.	Bonferroni
	Young-old (0.80)	Old (1.78)	−0.97	0.09	
		Old-old (1.94)	−1.14	0.04*	High > Low
	Old	Old-old	−0.17	1.00	

* *p* <.05.

## 3 Discussion

The present study is an initial investigation of changes in cognitive function scores of older community-dwelling Korean adults after participating in the Silvia program and of any differences among three age groups. The results provide evidence that the mobile-based multidomain program can contribute to an improvement in older Korean adults’ cognitive functioning. In addition, a comparison of the pre-post changes in cognitive function scores across the three different age groups indicated that the Old-old group demonstrated more change in cognitive functioning than the other age groups. Based on these two findings, we conclude that the mobile-based multi-domain program can be effective in supporting cognitive functioning, especially for the Old-old group.

Previous investigations of the effectiveness of single domain interventions, including exercise, cardiovascular health measures, diet, and cognitive training, have yielded positive outcomes among community-dwelling older adults (Brown et al., 2019, Hafdi et al., 2021, Liao et al., 2020). However, when deployed in large randomized clinical trials, many of these single-domain interventions have yielded mixed outcomes with regard to their effectiveness (Chow et al., 2002, Guo et al., 2020, National Institutes of Health, 2017, NIH RePORTER, 2017). The results of the present study support the effectiveness of a multi-domain intervention for preventing or reducing cognitive decline among older adults. This study adds to the body of knowledge evidence that the multi-domain intervention can support or improved cognitive functions of older adults (Lehtisalo et al., 2022, Ngandu et al., 2015).

Mobile-based applications are widely used by older adults to access information and stay connected with others (The Nielsen Company, 2018). Studies of the use of mobile applications for the purpose of dementia prevention have provided evidence that they can enable older adults to engage in various cognitive exercises resulting in reduction or prevention of cognitive decline (Eggink et al., 2021, Hafdi et al., 2021, Richard et al., 2019). The present study extends the body of knowledge by showing that a mobile-based multi-domain intervention that provides cognitive training, home-based exercise, AI evaluation, and other activity monitoring can lead to an improvement of older Korean adults’ cognitive functioning. This finding is particularly important in light of the WHO’s emphasis on testing the feasibility and acceptability of multi-domain programs for geographically and culturally diverse older adults. The focus on Korean older adults in this study expands the scope of mobile multi-domain applications for dementia prevention to older adults living in East Asia.

Prior studies have stressed the importance of cognitive activities, including engaging in computer/mobile-based games and cognitive exercises, for promoting cognitive functions (Stern and Munn, 2010, Yates et al., 2016). Other studies support the positive association between cognitive activities and cognitive performance including a reduced risk of dementia (Marquine et al., 2012, Sposito et al., 2015, Verghese et al., 2003). One of the main components that the Silvia program provided was cognitive training in various domains. The degree of difficulty of each program could be adjusted for each participant as the innovative AI evaluation measured the difficulties of each task, scores, time spent, and other relevant information for each participant as feedback for designing further activities. This mobile-based cognitive exercise component may be particularly supportive of users’ improvements in cognitive functioning. Researchers have also found evidence that physical activity/exercise programs can play an important role in improving the cognitive health of older adults and reducing their risk of dementia (Blondell et al., 2014, Guure et al., 2017, Rosness et al., 2014). By participating in physical activities, older adults can reduce the risks of developing Alzheimer’s disease and related dementias (ADRD) (Buchman et al., 2019, Palta et al., 2019). Much research has addressed the design and implementation of a variety of effective home-based PA programs and presented evidence that they can reduce depression and anxiety among older adults (Aguinaga et al., 2018). Since the onset of the COVID-19 pandemic, home-based PA programs have been strongly encouraged as a means to reduce mental health problems and concerns among older adults. The present study supports mobile-based home-exercise programs as an intervention to help prevent dementia among older adults.

While the FINGER and related studies reported clinical efficacy of digital healthcare for cognitive improvement, there has been controversy regarding age as a factor in understanding the different levels of the complementary effects of aging (Kivipelto et al., 2018a, Kivipelto et al., 2018b, Lehtisalo et al., 2022, Ngandu et al., 2015). A recent clinical study investigated the relationship between age and digital health usability and found that getting older is associated with a decrease in the number of completed performance tasks and an increase in the number of obstacles encountered (van der Vaart et al., 2019). It is worth noting, however, Kueider et al. (2012) reported the usability of technology-based activity is likely diminished for older populations, but the complementary effects of cognitive function still are associated with improved cognitive capability regardless of age. On the other hand, Kvavilashvlli et al. (2009) reported young-old adults are more likely to report difficulties with prospective memory (remembering to do something in the future) are than old-old individuals due to differences in the way that the two groups use different memory retrieval mechanisms. In addition to supporting that there is a complex relationship between different old stages and cognitive functions, the current study expands the findings of Kvavilashvili et al.’s study that the old-old group may use different memory retrieval mechanisms than the other two groups and experiences better cognitive function than they do even though our program focused on several aspects of cognitive domains.

Also, there is another possibility that Young-old participants has ceiling effects which may have reached high score on their baseline as the Table 1 indicated, the group of Young-old group received 28.55 which is the highest scores among the other age groups, while Old-old group received 24.06 at their baseline. Thus, we assumed that there is no room for demonstrating the improvements of this group and may consider there is a smaller improvement compared to other groups.

This study has some limitations that should be addressed in future research. The main limitations are related the single-arm design with no control group and a lack of follow-up data. Further studies using a randomized controlled trial across longer follow-up are recommended. In addition, in the present study we did not explore the relative effectiveness among the four domains (monitoring lifestyle, diet, home-based exercise programs, and cognitive training) for promoting healthy cognitive functioning. Future researchers might investigate the level of contribution of each domain to participants’ cognitive functioning.

Despite these limitations, in this study the effects of a mobile-based multi-domain program on older adults’ cognitive functioning were tested. Overall, this study confirms the importance of a mobile-based multi-domain intervention for dementia prevention. Based on the findings of this study, we conclude that designing and implementing a variety of mobile-based multi-domain platforms would contribute to supporting and improving the cognitive functions of older adults.

## Funding

This research has received funding from the Silvia Health Inc.

## Declaration of Competing Interest

The authors declare that they have no known competing financial interests or personal relationships that could have appeared to influence the work reported in this paper.

## Data Availability

Data will be made available on request.
